# Out-of-Sample Validity of the PROLOGUE Score to Predict Neurologic Function after Cardiac Arrest

**DOI:** 10.3390/jpm12060876

**Published:** 2022-05-26

**Authors:** Christoph Schriefl, Christian Schoergenhofer, Nina Buchtele, Matthias Mueller, Michael Poppe, Christian Clodi, Florian Ettl, Anne Merrelaar, Magdalena Sophie Boegl, Philipp Steininger, Michael Holzer, Harald Herkner, Michael Schwameis

**Affiliations:** 1 Department of Emergency Medicine, Medical University of Vienna, 1090 Vienna, Austria; christoph.schriefl@meduniwien.ac.at (C.S.); matthias.mueller@meduniwien.ac.at (M.M.); michael.poppe@meduniwien.ac.at (M.P.); christian.clodi@meduniwien.ac.at (C.C.); florian.ettl@meduniwien.ac.at (F.E.); anne.merrelaar@meduniwien.ac.at (A.M.); magda.boegl@gmail.com (M.S.B.); michael.holzer@meduniwien.ac.at (M.H.); michael.schwameis@meduniwien.ac.at (M.S.); 2 Department of Clinical Pharmacology, Medical University of Vienna, 1090 Vienna, Austria; christian.schoergenhofer@meduniwien.ac.at; 3 Department of Medicine I, Medical University of Vienna, 1090 Vienna, Austria; nina.buchtele@meduniwien.ac.at; 4 Emergency Department, Clinic Hietzing, Vienna Healthcare Group, 1130 Vienna, Austria; philipp.s@gmx.at

**Keywords:** cardiac arrest, risk estimation, prognostic score, external validation, independent sample, calibration, discrimination, data imputation

## Abstract

Background: The clinical value of a prognostic score depends on its out-of-sample validity because inaccurate outcome prediction can be not only useless but potentially fatal. We aimed to evaluate the out-of-sample validity of a recently developed and highly accurate Korean prognostic score for predicting neurologic outcome after cardiac arrest in an independent, plausibly related sample of European cardiac arrest survivors. Methods: Analysis of data from a European cardiac arrest center, certified in compliance with the specifications of the German Council for Resuscitation. The study sample included adults with nontraumatic out-of-hospital cardiac arrest admitted between 2013 and 2018. Exposure was the PROgnostication using LOGistic regression model for Unselected adult cardiac arrest patients in the Early stages (PROLOGUE) score, including 12 clinical variables readily available at hospital admission. The outcome was poor 30-day neurologic function, as assessed using the cerebral performance category scale. The risk of a poor outcome was calculated using the PROLOGUE score regression equation. Predicted risk deciles were compared to observed outcome estimates in a complete-case analysis, a best-case analysis, and a multiple-data-imputation analysis using the Markov chain Monte Carlo method. Results: A total of 1051 patients (median 61 years, IQR 50–71; 29% female) were analyzed. A total of 808 patients (77%) were included in the complete-case analysis. The PROLOGUE score overestimated the risk of poor neurologic outcomes in the range of 40% to 100% predicted risk, involving 63% of patients. The model fit did not improve after missing data imputation. Conclusions: In a plausibly related sample of European cardiac arrest survivors, risk prediction by the PROLOGUE score was largely too pessimistic and failed to replicate the high accuracy found in the original study. Using the PROLOGUE score as an example, this study highlights the compelling need for independent validation of a proposed prognostic score to prevent potentially fatal mispredictions.

## 1. Introduction

In cardiac arrest, there is a constant interest in early neurologic outcome prediction to aid health care professionals in providing appropriate care to patients and valid information to relatives.

However, only the minority of prediction scores developed for clinical use are eventually implemented in daily practice [1] because the clinical value of a given prediction score relies on its out-of-sample validity (i.e., its transportability to different locations, settings, and populations).

The very recently developed PROLOGUE prediction score (PROgnostication using LOGistic regression model for Unselected adult cardiac arrest patients in the Early stages) accurately discriminated neurologic outcome at hospital discharge in a large Korean cohort of unselected cardiac arrest survivors (area under the curve [AUC] 0.94) [2]. The score is attractive, as it can be used in both in- and out-of-hospital cardiac arrest cases, in witnessed and unwitnessed events, and independent of the initial cardiac rhythm or whether targeted temperature management was applied or not. The score does not require knowledge of no-flow duration, which is commonly unknown, but it uses dichotomized data readily available at hospital admission, translating into a high score practicability.

We assessed the validity of the PROLOGUE score in an independent sample of European cardiac arrest survivors.

## 2. Materials and Methods

### 2.1. Study Design, and Setting

We analyzed data from the Vienna Clinical Cardiac Arrest Registry, which prospectively includes all adult cardiac arrest patients admitted to and treated at the Department of Emergency Medicine at the Medical University of Vienna, a cardiac arrest center certified in compliance with the specifications of the German Council for Resuscitation and a member of the Extracorporeal Life Support Organization. Data acquisition and documentation were conducted in accordance with Utstein-style guidelines for cardiac arrest-related documentation [3]. Reporting follows the TRIPOD statement.

### 2.2. Study Population

Patient selection was based on the eligibility criteria applied by Dae Hee Bae and colleagues [2]. Adults (≥18 years) who experienced nontraumatic in- or out-of-hospital cardiac arrest and achieved sustained return of spontaneous circulation (ROSC) between January 2013 and December 2018 were eligible. Patients with hemorrhagic or ischemic stroke after ROSC or a prearrest cerebral performance category (CPC) >2 were excluded from study participation.

### 2.3. Outcome

The outcome was neurologic function 30 days after ROSC, which was assessed by study fellows using the five-point CPC scale, as described previously [4]. A good neurologic outcome was defined as CPC 1 (full recovery) or 2 (moderate disability). Poor neurologic outcome was defined as CPC 3 to 5 (severe disability, vegetative state, or death, respectively) or persistent unresponsiveness due to analgosedation during the study period or before death, in accordance with the Utstein-style guidelines [3]. Outcome data were available for all study patients.

### 2.4. Statistical Analysis

We present categorical data as absolute numbers and relative frequencies, and continuous data as the mean with standard deviation (±SD) or the median with 25–75% interquartile range (IQR). Outcome estimates are presented as proportions with 95% confidence intervals (CIs).

We calculated the deciles of poor outcome risk using the multivariable regression equation of the PROLOGUE model e^βX^/(1 + e^βX^), where e = 2.7182818, and βX = 6.261 + (−0.515 if witnessed collapse) + (−1.087 if shockable rhythm) + (−1.158 if reactive pupillary light reflex) + (−1.304 if age < 59 years) + (−0.67 if adrenaline dose < 2 mg) + (−0.745 if low-flow duration < 18 min) + (−0.83 if creatinine < 1.21 mg dL^−1^) + (−0.557 if potassium < 4.4 mEq L^−1^) + (−0.838 if phosphate < 5.8 mg dL^−1^) + (−0.813 if hemoglobin ≥ 13.2 g dL^−1^) + (−0.63 if lactate < 8 mmol L^−1^) + (−1.671 if GCS motor score ≥ 2). Predicted risk deciles were then compared to observed outcome estimates with 95% confidence intervals (95% CI) in a complete case analysis, including only patients in whom complete information on predictors and outcome was available, a best-case analysis (counting missing predictor values as zero), and a multiple data imputation analysis using a Markov chain Monte Carlo method, assuming that all variables in the model have a joint multivariate normal distribution. The data augmentation algorithm imputes missing data by drawing from a multivariate normal data distribution, given the observed data. Given a sufficient sample size, a multivariate normal distribution provides reliable estimates even when the normality assumption is violated, but biased estimates can be observed in the case of highly missing information [5,6].

The Hosmer–Lemeshow test was used to assess model fit, i.e., the match between predicted and observed event rates. Receiver operating characteristics (ROC) analysis was performed to assess model discrimination, presented as AUC with 95% CIs [7]. Fisher’s exact test and Student’s *t*-test were used to compare predictor variables and event rates between the PROLOGUE and the current study sample. We used Stata Statistical Software (Release 17, StataCorp. 2021, StataCorp LLC, College Station, TX, USA) for data analysis. A two-sided *p* value < 0.05 was considered statistically significant.

## 3. Results

Of the 1591 cardiac arrest patients enrolled in the registry during the observation period, 1051 patients (median age 61 years, IQR 50–71; 29% female) met the eligibility criteria and were further analyzed. Patient characteristics according to outcome are presented in Table 1. Overall, 55% of patients (578/1051) had a poor neurologic outcome (CPC 3–5) on day 30. Complete information on predictor variables and outcomes was available for 808 patients (76.9%) who were included in the complete case analysis.

Table 2 shows comparisons of predictor variables and outcome event rates between the study sample and the PROLOGUE derivation sample.

**Figure 1:**
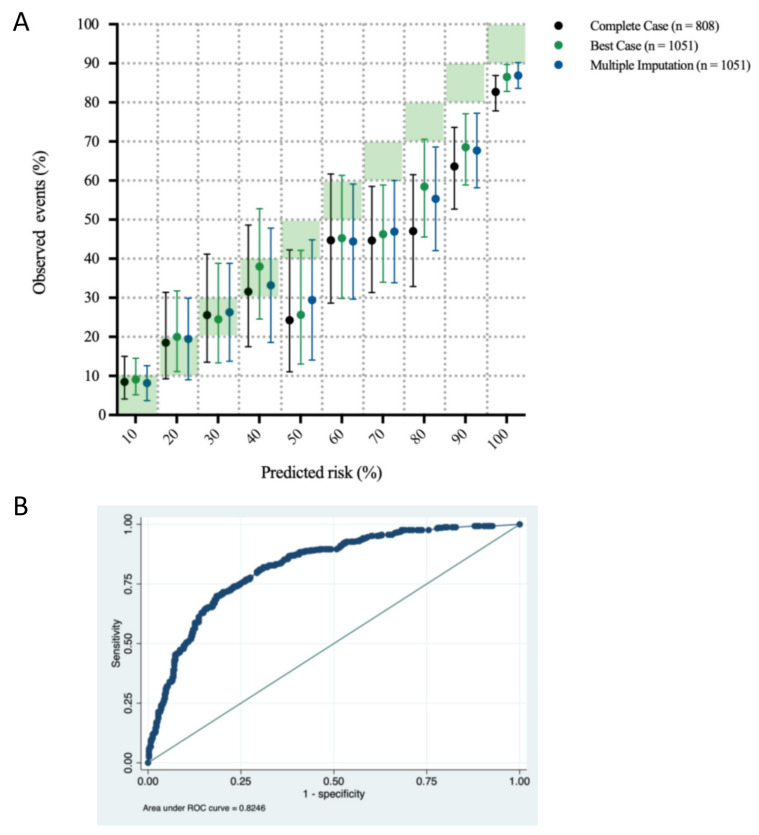
(**A**) Observed event point estimates with 95% CIs by predicted risk deciles of poor neurologic outcomes. X-axis: Risk deciles as predicted by the PROLOGUE score; Y-axis: Observed event estimates with 95% CI (bars) for the complete case analysis (Hosmer–Lemeshow test *p* = 0.0024), and after best case (Hosmer–Lemeshow test *p* = 0.0058) and multiple data imputation (Hosmer–Lemeshow test *p* = 0.0122). (**B**) ROC curve for the PROLOGUE score applied to the complete case dataset. X-axis: 1-specificity; Y-axis: Sensitivity. Abbreviation: ROC curve, Receiver Operating Characteristic curve.

Figure 1 shows observed event rates across deciles of the predicted risk of poor neurologic outcome and the ROC curve for outcome prediction by applying the PROLOGUE score to the complete case dataset. The PROLOGUE score overestimated the risk of poor neurologic outcome between a 40% and 100% predicted risk, involving 63% (complete case) of patients. The AUC for the PROLOGUE score was 0.82 (95% CI 0.80 to 0.85).

Best case and multiple data imputation did not improve the model fit.

## 4. Discussion

This study aimed to validate the PROLOGUE score using a sample from a large independent database of European cardiac arrest patients. The PROLOGUE score did not have the accuracy reported in the original study (AUC 0.94) [2] and overestimated the risk of poor neurologic outcomes in the majority of cardiac arrest survivors.

The number of publications identified by the search terms ‘cardiac arrest and prediction’ on PubMed has substantially increased over the past decades, from 34 in 1989 to 648 in 2020. The ongoing tremendous effort put into prediction research highlights the unmet medical need for accurate outcome estimation after successful resuscitation. However, the majority of scores developed for this purpose are never validated independently or used in clinical care.

The PROLOGUE score appears attractive for clinical implementation because it was developed and internally validated on a large heterogeneous patient sample. The PROLOGUE sample represents an unselected ‘real-world’ population on whom a prognostic score would effectively be used in clinical practice. It does not require knowledge on no-flow times or bystander CPR but includes variables readily available at hospital admission with presumably homogenous effects across varying populations. By applying the same eligibility criteria, we analyzed a plausibly related European patient sample with a 55% event rate.

However, despite the similar patient age range and duration of CPR, the distribution of predictor values largely differed between the two samples. The proportions of cardiac etiology and shockable rhythm were lower in the PROLOGUE sample. Given the similar atherosclerotic CVD prevalence in Korea and Europe [8], this may suggest differences in prehospital resuscitation policies or in ‘true’ no-flow times between the samples [9,10].

These differences in case mix, despite a similarly defined target population, underline the importance of out-of-sample score validity assessment. Although a patient cohort is defined similarly to a study cohort in which a particular prognostic score is developed in terms of eligibility criteria, study window size, and definitions of predictor variables and outcomes, the performance of the score may be strongly influenced by different patient characteristics. Likewise, Dae Hee Bae et al. developed a score on a complete case sample, which usually provides biased estimates. Our findings highlight the significance of recognizing the sample on which a score was developed to assess its applicability.

The need for early risk stratification in cardiac arrest will further increase, given the increasing availability of extracorporeal resuscitation modalities and related decisions on resource-intensive treatment escalation or withdrawal due to futility. Inaccurate outcome estimates may not only be useless but also potentially fatal.

### Limitations

In the current study, we analyzed data from the Vienna Cardiac Arrest Registry, a sample of central European cardiac arrest patients. However, although we analyzed a large sample, we used data from a single center, and we cannot rule out that local resuscitation policies limit the representativeness of our study cohort. Furthermore, we used 30-day neurologic function as an outcome, which may differ from the neurologic function at hospital discharge used in the original study by Dae Hee Bae et al. However, the 33-day median length of hospital stay of cardiac arrest survivors at our institution suggests no substantial differences between the outcome variables. In addition, the potential heterogeneity of predictor measurements may affect out-of-sample performance estimates but was not assessed. Moreover, it should be noted that we did not examine the performance of the PROLOGUE score in a sample that differed from the definition of the original target population. For example, whether the performance of the score also depends on the gender of the patients cannot be said on the basis of our analysis because gender was not a predictor of the original PROLOGUE regression model, and the PROLOGUE score is intended to be applied regardless of gender.

Furthermore, the complete case analysis included 76% of all study patients, which may be a source of bias. However, the two common methods of data imputation did not improve the model fit. In this context, it should be noted that the original PROLOGUE score was based on a complete case analysis without data imputation, excluding almost 20% of patients, which may contribute to its limited out-of-sample validity. Finally, it should be noted that the assessment of outcomes in cardiac arrest must always be multimodal, never based on a single parameter or score, and, according to the current European Resuscitation Council guidelines 2021, not performed before 72 h after cardiac arrest [11].

## 5. Conclusions

This independent validation of the PROLOGUE score in a sample of European cardiac arrest survivors failed to replicate the high accuracy found in the original study. Our results underline the importance of differences in patient characteristics between plausibly related populations and highlight the need for external score validation to eventually foster the implementation of prediction scores in clinical practice and to avoid inaccurate predictions.

## Figures and Tables

**Table 1:** Characteristics of study patients according to neurologic outcome on day 30.

Variable	Total N = 1051	Good Neurologic Outcome N = 473	Poor Neurologic Outcome N = 578
Age, median (IQR)	61 (50–71)	58 (48–68)	64 (53–74)
Female, *n* (%)	303 (29)	126 (27)	177 (31)
Chronic health conditions, *n* (%)			
Diabetes	214 (20)	78 (16)	136 (24)
Hypertension	470 (45)	189 (40)	281 (49)
Current smoker	313 (30)	162 (34)	151 (26)
Chronic heart failure	137 (13)	46 (10)	91 (16)
Myocardial infarction	115 (11)	55 (12)	60 (10)
Cerebral vascular insufficiency	74 (7)	22 (5)	52 (9)
Coronary artery disease	226 (22)	94 (20)	132 (23)
Chronic obstructive pulmonary disease	130 (12)	40 (8)	90 (16)
Out-of-hospital cardiac arrest, *n* (%)	833 (79)	365 (77)	468 (81)
Witnessed, *n* (%)	921 (88)	437 (92)	484 (84)
Bystander CPR, *n* (%)	545 (52)	268 (57)	277 (48)
Initial shockable rhythm, *n* (%)	593 (56)	341 (72)	252 (44)
No-Flow (min), median (IQR)	0 (0–0)	0 (0–0)	0 (0–0)
Low-Flow (min), median (IQR)	19 (9–36)	13 (4–23)	27 (15–48)
Total adrenaline (mg), median (IQR)	3 (1–5)	2 (1–4)	3 (2–6)
Number of shocks applied, median (IQR)	3 (1–5)	2 (1–4)	3 (1–7)
Cause of cardiac arrest, *n* (%)			
Pulmonary	145 (14)	38 (8)	107 (19)
Cardiac	744 (71)	378 (80)	366 (63)
Metabolic	11 (1)	3 (1)	8 (1)
Intoxication	21 (2)	5 (1)	16 (3)
Drowning	1 (0)	1 (0)	0 (0)
Sepsis	10 (1)	3 (1)	7 (1)
Cerebral	5 (0)	0 (0)	5 (1)
Other	36 (3)	12 (3)	24 (4)
Unknown	78 (7)	33 (7)	45 (8)
Laboratory values after ROSC, median (IQR)			
pH	7.17 (7.03–7.28)	7.26 (7.15–7.33)	7.11 (6.95–7.22)
Lactate (mmol/L)	6.9 (4.1–10.5)	4.9 (2.9–7.6)	8.7 (5.8–12.1)
Hemoglobin (g/dL)	13.5 (11.9–14.7)	14.1 (12.7–15.0)	13.0 (11.1–14.4)
C-reactive protein (mg/dL)	0.37 (0.15–1.23)	0.28 (0.11–0.72)	0.50 (0.17–1.86)
Creatinine (mg/dL)	1.24 (1.00–1.55)	1.11 (0.90–1.35)	1.39 (1.11–1.76)
Blood urea nitrogen (mg/dL)	17.1 (13.0–22.5)	15.6 (12.6–20.2)	18.4 (13.8–25.9)
Albumin (g/dL)	36.7 (32.6–39.8)	38.5 (35.0–41.1)	35.0 (30.6–38.1)
Bilirubin (mg/dL)	0.40 (0.27–0.67	0.42 (0.27–0.68)	0.40 (0.26–0.66)
Glucose (mg/dL)	241 (171–319)	213 (160–285)	275 (193–345)
Potassium (mmol/L)	3.93 (3.53–4.48)	3.78 (3.42–4.21)	4.10 (3.64–4.74)
Sodium (mmol/L)	139 (136–141)	139 (137–141)	138 (136–141)
Chloride (mmol/L)	100 (97–103)	101 (98–103)	99 (96–102)
Magnesium (mmol/L)	0.93 (0.84–1.11)	0.89 (0.81–0.97)	1.01 (0.89–1.18)
Phosphate (mg/dL)	5.98 (4.27–8.33)	4.71 (3.50–6.32)	7.50 (5.54–9.38)
Temperature after ROSC (°C), median (IQR)	35.6 (34.9–36.2)	35.7 (35.1–36.3)	35.4 (34.5–36.2)
GCS after ROSC, median (IQR)	3 (3–5)	3 (3–15)	3 (3–3)
GCS motor score after ROSC, median (IQR)	1 (1–2)	1 (1–6)	1 (1–1)
Reactive pupillary light reflex, *n* (%)	503 (48)	324 (68)	179 (31)
ECMO, *n* (%)	84 (8)	33 (7)	51 (9)
Targeted temperature management, *n* (%)	738 (70)	297 (63)	441 (76)

Good neurologic outcome was defined as CPC 1–2, poor neurologic outcome as CPC 3–5. Abbreviations: CPC, Cerebral Performance Category; CPR, Cardiopulmonary Resuscitation; ECMO, extracorporeal membrane oxygenation; GCS, Glasgow Coma Scale; ROSC, Return of Spontaneous Circulation.

**Table 2:** Comparisons of predictor variables and event rates between the study sample and the PROLOGUE derivation sample.

	Study Sample (N = 1051)	PROLOGUE Sample (N = 671)	*p*-Value
**Predictor Variables**			
Age, years; mean (SD)	61 (16)	61 (16)	1
GCS motor score; mean (SD)	1 (1)	2.5 (2)	<0.001
Adrenaline, mg; mean (SD)	3 (3)	2 (2.5)	<0.001
Low-flow time, min; mean (SD)	19 (20)	20 (14)	0.2584
Creatinine, mg/dL; mean (SD)	1.24 (0.41)	1.25 (0.52)	0.6573
Potassium, mmol/L; mean (SD)	3.93 (0.70)	4.2 (0.92)	<0.001
Phosphate, mg/dL; mean (SD)	5.98 (3.0)	6.0 (2.14)	0.8808
Hemoglobin, g/dL; mean (SD)	13.5 (2.07)	12.7 (2.66)	<0.001
Lactate, mmol/L; mean (SD)	6.9 (4.74)	9.45 (4.51)	<0.001
Witnessed collapse, *n* (%)	921 (88)	460 (69)	<0.001
Shockable rhythm, *n* (%)	593 (56)	203 (30)	<0.001
Reactive pupillary reflex, *n* (%)	503 (48)	367 (55)	0.0065
**Poor Outcome (CPC 3–5); *n* (%)**	578 (55) **^#^**	443 (66) **^+^**	<0.001

**^#^** 30 days after return of spontaneous resuscitation; **^+^** at hospital discharge. Abbreviations: CPC, Cerebral Performance Category; GCS, Glasgow Coma Scale.

## Data Availability

The datasets used and analyzed during the current study are available from the corresponding author on reasonable request.
